# Reliability and responsiveness of social connection in long‐term care home residents (SONNET) scale

**DOI:** 10.1002/alz70861_108812

**Published:** 2025-12-23

**Authors:** Neha Dewan, Jennifer Bethell, Madalena Liougas, Andrew Sommerlad

**Affiliations:** ^1^ Western University of Health Sciences, Lebanon, OR USA; ^2^ University of Toronto, Toronto, ON Canada; ^3^ ICES, Toronto, ON Canada; ^4^ KITE Research Institute, Toronto Rehabilitation Institute ‐ University Health Network, Toronto, ON Canada; ^5^ North London NHS Foundation Trust, London, London UK; ^6^ University College London, London, London UK

## Abstract

**Background:**

Social connection is important for health and quality of life in long‐term‐care (LTC) settings, particularly for long term care residents with dementia. However, the field lacks a psychometrically sound tool to measure social connection in a reliable and responsive manner. The Social Connection in Long Term Care Residents (SONNET) scale was recently developed by our team to fill this gap. We have established internal consistency and construct validity for the SONNET scale, yet its reproducibility and responsiveness across time and populations remains to be explored. This study aims to evaluate the test‐retest reliability and responsiveness of the SONNET scale among residents of LTC homes located in rural communities, with a focus on those with cognitive impairment, to determine its utility as an outcome measure in intervention studies.

**Methods:**

A prospective, multi‐site study will be conducted in LTC settings in Oregon, US. Residents aged 65+ with mild to moderate dementia will be recruited. The SONNET scale will be administered at baseline and after a 3‐day interval to assess test‐retest reliability. To evaluate responsiveness, a subgroup of participants will engage in a structured intervention to improve social connection, with SONNET administered before and after the intervention period at 3 and 6 months. Floor and ceiling effects will be calculated and considered to exist if >15% of participants scored minimal or maximal scores as their total scores, respectively. Intraclass correlation coefficients (ICC) will be used to assess relative reliability, standard error measurement, minimal detectable change and bland Altman plots will be used to evaluate absolute reliability. Effect size (ES), Standardized response means (SRM), and minimal clinically important difference will be calculated to evaluate responsiveness.

**Results:**

We anticipate that the SONNET scale will demonstrate good test‐retest reliability (ICC ≥ 0.70) and moderate to high responsiveness (ES ≥ 0.50; SRM ≥ 0.50) in detecting changes in social connection following the intervention.

**Conclusion:**

Establishing the reproducibility and responsiveness of the SONNET scale will support its use in both research and practice, providing a robust tool for assessing social connection and informing interventions to improve well‐being among LTC residents with dementia.

